# Mott Memorial Library

**Published:** 1867-01

**Authors:** 


*Mott Memorial Library.*—Mrs. Mott has made a noble contribution to the profession of New York, at the same time that she has erected a perpetual monument to the memory of her husband, the late distinguished VALENTINE MOTT. We take the following account from the *Philadelphia Reporter:*

What the late Professor Mutter did for Philadelphia, the widow of the late Professor Valentine Mott has done for New York. At an expense of more than $30,000, she has purchased, enlarged, and fitted up, at No. 58, Madison Avenue, between 27th and 28th streets, a building, in which are deposted the medical library, and the surgical instruments of her late husband, the distinguished American surgeon, Valentine Mott.—*Cincinnati Lancet and Observer.*

